# Ferromagnetic 2p-2p and 4f-2p Couplings in a Macrocycle from Two Biradicals and Two Gadolinium(III) Ions

**DOI:** 10.3390/molecules27154930

**Published:** 2022-08-02

**Authors:** Saki Ito, Toru Yoshitake, Takayuki Ishida

**Affiliations:** Department of Engineering Science, The University of Electro-Communications, Chofu, Tokyo 182-8585, Japan; ito@ttf.pc.uec.ac.jp (S.I.); yoshitake@ttf.pc.uec.ac.jp (T.Y.)

**Keywords:** high spin, exchange interaction, magneto-structural relation, aminoxyl

## Abstract

A new ground triplet biradical 2′,4′,6′-triisopropylbiphenyl-3,5-diyl bis(*tert*-butyl nitroxide) (**iPr_3_BPBN**) was prepared and characterized by means of room-temperature ESR spectroscopy displaying a zero-field splitting pattern together with a half-field signal. Complex formation with gadolinium(III) 1,1,1,5,5,5-hexafluoropentane-2,4-dionate (hfac) afforded a macrocycle [{Gd(hfac)_3_(μ-**iPr_3_BPBN**)}_2_]. As the X-ray crystallographic analysis clarified, the biradical serves as a bridging ligand, giving a 16-membered ring, where each nitroxide radical oxygen atom is directly bonded to a Gd^3+^ ion. The magnetic study revealed that the **iPr_3_BPBN** bridge behaved as a practically triplet biradical and that the Gd^3+^-radical magnetic coupling was weakly ferromagnetic. The exchange parameters were estimated as 2*j*_rad-rad_/*k*_B_ > 300 K and 2*J*_Gd-rad_/*k*_B_ = 1.2 K in the *H* = −2*J* *S*_1_•*S*_2_ convention. The DFT calculation based on the atomic coordinates clarified the ground triplet nature in metal-free **iPr_3_BPBN** and the enhanced triplet character upon coordination. The calculation also suggests that ferromagnetic coupling would be favorable when the Gd-O-N-C(sp^2^) torsion comes around 100°. The present results are compatible with the proposed magneto-structure relationship on the nitroxide-Gd compounds.

## 1. Introduction

**Scheme 1:**
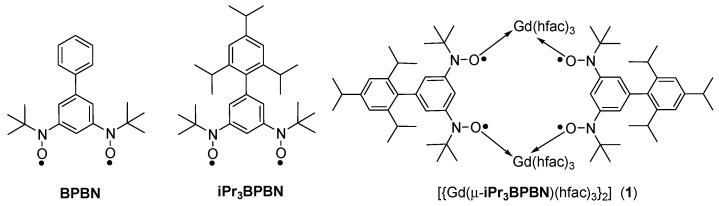
Molecular structures of **BPBN**, **iPr_3_BPBN**, and **1**.

Lanthanide(4f)-radical(2p) heterospin systems afford an important class of magnetic materials [1,2,3,4,5,6]. As a 2p spin source, nitroxide or aminoxyl radicals [7] are widely demanded, because the oxygen atom of nitroxide radicals can serve as an O-donor to metal ions [4,5,6,7,8,9,10,11], and 4f-2p magnetic exchange couplings are operational [12,13,14,15,16]. Exchange coupling will reduce possible quantum tunneling of magnetization in single-molecule magnets [5,17,18]. Accordingly, evaluation of exchange coupling is thus vital in the study of molecule-based magnets. To develop one- or higher-dimensional structures toward synthetic magnets, bisnitroxide radicals seem to be attractive. When they behave as a bridging ligand between 4f ions, the bridge must be a paramagnetic molecule. To apply ground triplet biradicals to materials chemistry, the choice of stable radicals is crucial. It has been theoretically and experimentally clarified that 1,3-phenylene-bridged oligoradicals would have a ground high-spin state in the context of the spin-polarization mechanism [19,20,21,22,23,24]. The biphenyl-3,5-diyl bis(*tert*-butyl nitroxide) (**BPBN**) biradicals [25] (Scheme 1) having enough persistency to be isolated under ambient conditions seem to be promising in the study of 4f-2p heterospin magnetism. 

**Scheme 2:**
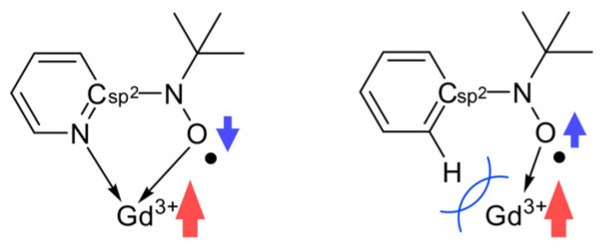
Typical structure-dependence of Gd-nitroxide exchange coupling.

Ferromagnetic 4f-2p exchange coupling often occurs across coordination bonds, and the nature, ferro- or antiferromagnetic, and their magnitude depend on the coordination structure [13,14,15,16]. A gadolinium(III) ion is often selected as a first choice to investigate exchange coupling in a spin-only treatment according to conventional magnetometry. The Gd-nitroxide magneto-structural relationship has been proposed, where the π-conjugated nitroxide radicals are incorporated (Scheme 2). The in-plane location of a Gd^3+^ ion on the radical π* node would favor antiferromagnetic coupling, whereas dislocation from the nodal plane prefers a ferromagnetic one [26,27]. The plausible mechanism for the ferromagnetic 4f-2p coupling model is reviewed briefly. The presence of 5d(Gd^3+^)–2p*z*(O_rad_) overlap is beneficial to charge transfer (CT), and the high-spin state in a Gd ion (4f and 5d orbitals) is stabilized by Hund’s rule. The ground and CT states are admixed and, consequently, high-spin configuration is stabilized, or ferromagnetic interaction is observed. Molecular designs have been successful by using 2-pyridyl and phenyl substituents [14,16,28,29,30]. During the study using phenyl nitroxide ligands, we noticed that the strong O-donating ability can afford Gd-nitroxide coordination compounds without any chelate support [30,31]. 

We take advantage of the steric effect from substituents on the peripheral phenyl group in **BPBN** [14,15] to avoid 4f-2p antiferromagnetic coupling taking place after the chelation. Here, a new triisopropylated biradical, **iPr_3_BPBN** (Scheme 1), has been designed for the present purpose. Eventually, [{Gd(hfac)_3_(μ-**iPr_3_BPBN**)}_2_] (**1**) was synthesized as a novel 4f-2p heterospin system.

## 2. Materials and Methods

### 2.1. Preparation of New Compounds

According to the method known for the preparation of a family of **BPBN** radicals decorated with substituents [32,33,34], the Suzuki coupling reaction was conducted from 3,5-bis(*N*-*tert*-butylhydroxylamino)bromobenzene (0.455 g; 1.38 mmol) and 2,4,6-triisopropylphenyl boronic acid (0.384 g; 1.55 mmol) in the presence of Pd(PPh_3_)_4_ (85 mg; 0.070 mmol) and Na_2_CO_3_ (0.454 mg; 4.28 mmol) in dioxane (15 mL) under a nitrogen atmosphere. After the mixture was heated at 100 °C for 1 d, the reaction was quenched with an excess amount of water. After the organic layer was separated, washed, dried over anhydrous MgSO_4_, and concentrated under reduced pressure, the radical precursor was purified by a short column chromatography (silica gel eluted with hexane/ether) followed by recrystallization from dichloromethane/hexane. The yield was 0.324 g (52%). Mp. 229–232 °C. ^1^H NMR (500 MHz, DMSO-*d*_6_) *δ* 8.32 (s, 2H, OH), 7.20 (s, 1H, Ar-H), 7.02 (s, 2H, Ar-H), 6.59 (s, 2H, Ar-H), 2.89 (septet, 1H, iPr), 2.60 (septet, 2H, iPr), 1.23 (d, 6H, iPr), 1.08 (s, 18H, *t*-Bu), 1.02 (d, 12H, iPr). ^13^C NMR (126 MHz, DMSO-*d*_6_) *δ* 149.89, 147.37, 145.87, 137.56, 136.87, 121.50, 120.16, 118.57, 59.71, 33.69, 29.78, 26.09, 24.11, 24.10. MS (ESI^+^, MeOH) *m*/*z* 477 (M + Na^+^). IR (neat; attenuated total reflection (ATR)) 3213, 2958, 2929, 2868, 1585, 1479, 1458, 1360, 1231, 1199, 823, 720 cm^−1^. The above precursor was oxidized with freshly prepared Ag_2_O in dichloromethane. A red crystalline product of **iPr_3_BPBN** was purified by recrystallization from dichloromethane/hexane. The yield was 78%. Mp. 139–140 °C. The spectroscopic data are: ESR (9.84 GHz, toluene at room temperature) *g* = 2.0063, a peak-to-peak linewidth Δ*B*_pp_ = 2.2 mT. MS (ESI^+^, MeOH) *m*/*z* 475 (M + Na^+^). IR (neat; ATR) 2953, 2866, 1605, 1547, 1400, 1383, 1219, 1187, 857, 735 cm^−1^. Anal. Calcd. For C_29_H_44_N_2_O_2_: C, 76.95%; H, 9.80%; N, 6.19%. Found: C, 76.71%; H, 10.17%; N, 6.28%. Selected spectroscopic data are shown in Figures S1–S3, Supporting Information.

After a heptane solution (40 mL) containing [Gd(hfac)_3_(H_2_O)_2_] [35] (82.9 mg; 0.102 mmol) was refluxed and concentrated to ca. 10 mL, a CH_2_Cl_2_ solution (2 mL) of **iPr_3_BPBN** (49.9 mg; 0.11 mmol) was combined to the above solution at room temperature. After being allowed to stand in a freezer (–20 °C) for 3 d, orange needle-like crystals of [{Gd(hfac)_3_(μ-**iPr_3_BPBN**)}_2_]•(CH_2_Cl_2_)_2_ (abbreviated as **1**•(CH_2_Cl_2_)_2_ hereafter) were precipitated. They were collected, washed, and air-dried on a filter. The yield was 63.7 mg (51%). Mp. 98–99 °C. Anal. Calcd. for desolvated sample C_88_H_94_F_36_N_4_O_16_Gd_2_: C, 42.93%; H, 3.85%; N; 2.28%. Found: C, 42.97%; H, 3.85%; N: 2.37%. IR (neat, ATR): 2965, 1667, 1651, 1557, 1530, 1505, 1465, 1328, 1254, 1197, 1140, 1100, 880, 860, 797, 768, 741, 660, 586, 528, 467, 421 cm^−1^.

### 2.2. Instrumentation and Methods 

X-Ray diffraction data of **iPr_3_BPBN** and **1**•(CH_2_Cl_2_)_2_ were collected on a Saturn70 Hybrid Pixel Array Detector with graphite monochromated Mo Kα radiation (*λ* = 0.71073 Å). The structures were directly solved and expanded using Fourier techniques in the Olex2 1.3 and 1.5 programs [36]. The parameters were refined on SHELXL [37]. The solution of **iPr_3_BPBN** required a disorder model for the two-fold positions in the 4-isopropylphenyl portion. As for **1**•(CH_2_Cl_2_)_2_, hydrogen atoms were located at calculated positions, and their parameters were refined as “riding”. The space group was a tetragonal *I*4_1_/*acd*. A disorder was found for three hfac groups, and two crystallograhically independent units are superposed. Another possibility was an orthorhombic *Ibca* space group, where accidental *a* ≈ *b* was assumed and a half molecule was independent. A disorder model must be applied to three hfac groups, similarly to the case of the *I*4_1_/*acd* solution. Owing to possible parameter interaction, anisotropic refinement gave structural divergence. We conclude that *I*4_1_/*acd* would give a better optimized structure. Selected crystallographic data are listed in Table 1.

ESR spectra of **iPr_3_BPBN** were recorded on a Bruker ELEXYS X-band (9.4 GHz) spectrometer. The sample was dissolved in toluene and degassed with a nitrogen stream in a quartz tube. The concentration of the samples was ca. 1 × 10^−4^ mol L^−1^. For a film dispersion sample for solid-solution spectra was prepared by dissolving **iPr_3_BPBN** (1 mg) in chlorobenzene (1 mL) containing poly(vinyl chloride) (100 mg), followed by film casting on a glass plate. The spectrum simulation was employed in the WIN-EPR SimFonia version 1.2 program package [38]. 

The magnetic susceptibilities and magnetizations of **iPr_3_BPBN** and **1** were measured on a Quantum Design MPMS-XL7 SQUID magnetometer with static fields of 0.5 and 0.05 T, respectively. The magnetic responses were corrected with diamagnetic blank data of the sample holder measured separately. The diamagnetic contribution of the sample itself was estimated from Pascal’s constants [39]. 

Density-functional theory (DFT) calculations on **iPr_3_BPBN**, [{Y(hfac)_3_(H_2_O)}_2_(μ-**iPr_3_BPBN**)], and [Gd(hfac)_3_(H_2_O)(**iPr_3_BPBNH**)] were carried out on Gaussian16 Revision C.01 [40]. For the molecular structures, see the results section. The broken symmetry method [41,42,43] with the unrestricted B3LYP theory was applied to the basis sets of SDD (Stuttgart/Dresden quasi-relativistic effective core potentials) [44,45] for Gd, LANL2DZ for Y, and 6-311+G(d) for other elements. The self-consistent field energies were calculated with the experimentally determined coordinates. The exchange coupling parameter was reduced with Yamaguchi’s equation: *J* = (*E*_LS_ − *E*_HS_)/(<*S*^2^>_HS_ − <S^2^>_LS_) in the $\hat{H}$ = −2*J*$\hat{S}$_1_.$\hat{S}$_2_ convention [46].

## 3. Results and Discussion

### 3.1. Structure and Magnetic Properties of **iPr_3_BPBN**

**Figure 1:**
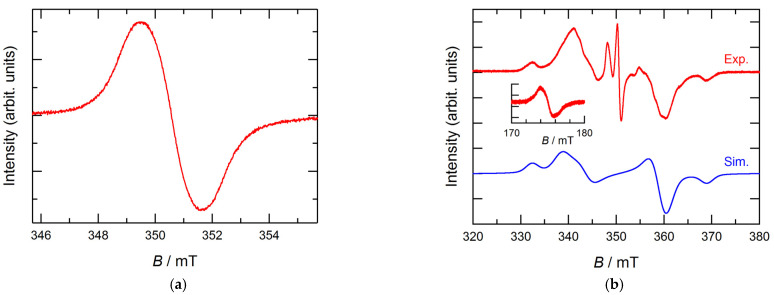
(**a**) X-Band ESR spectrum of **iPr_3_BPBN** in a toluene solution measured at room temperature. (**b**) Experimental (red) and simulated (blue) ESR spectra of a poly(vinyl chloride) dispersion film containing 1 w/w% of **iPr_3_BPBN**, measured at 292 K. Relatively sharp signals around 350 mT are ascribable to monoradical impurity. The inset shows a |Δ*m_S_*| = 2 forbidden signal at a half magnetic field.

At the first stage, the biradical ligand solely was structurally and magnetically characterized. A precursory bishydroxylamine was prepared according to the Suzuki coupling reaction as a key step from 3,5-bis(*N*-*tert*-butylhydroxylamino)bromobenzene and 2,4,6-triisopropylphenyl boronic acid. The oxidation of the precursor with Ag_2_O gave **iPr_3_BPBN**. The solution ESR spectroscopy (9.84 GHz, toluene at room temperature) clarified *g* = 2.0063 with a peak-to-peak linewidth Δ*B*_pp_ = 2.2 mT (Figure 1a). Such a broad single line is regarded as a fingerprint of the *m*-phenylene bisnitroxide family [25,32,33,34], which can be explained in terms of intramolecular dipolar coupling. The solid-solution ESR spectrum of **iPr_3_BPBN** in a poly(vinyl chloride) film displayed a typical fine structure together with a |Δ*m_S_*| = 2 forbidden transition at 175 mT as a half-field (Figure 1b). Note that these spectra were acquired at 292 K. This finding suggests the meaningful population of a triplet state even at room temperature. A point-dipole approximation showed that the distance between the spins was *r* = 5.3 Å from |2*D*|/*gμ*_B_ = 36.6 mT. The distance is consistent with the molecular structure of **iPr_3_BPBN**; the *r* value calculated is longer than the intramolecular N•••N, O•••O, and N•••O distances of 4.7940 (10), 4.6900 (10), and 4.8824 (10) Å, respectively, from the crystallographic analysis (see below). Therefore, in solution, the syn/anti conformation would be postulated, rather than the syn/syn conformation, where syn and anti nomenclatures are defined with respect to the C(sp^2^)-N single bond. 

**Figure 2:**
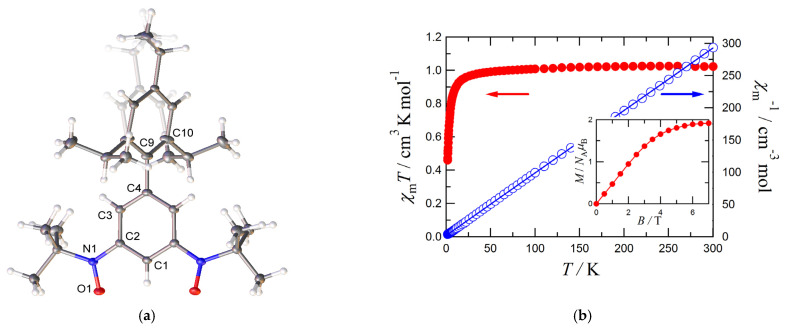
(**a**) X-ray crystal structure of **iPr_3_BPBN** with thermal ellipsoids at the 50% probability level for non-hydrogen atoms. A disordered position is shown with shaded atoms. A Selective atomic numbering is also shown. (**b**) Temperature dependence of *χ*_m_*T* and *χ*_m_^−1^ for **iPr_3_BPBN** measured at 0.5 T. A solid line stands for the Curie–Weiss analysis. Inset: the *M-H* curve of **iPr_3_BPBN** measured at 1.8 K.

Biradical **iPr_3_BPBN** crystallizes in a monoclinic *C*2/*c* space group with *Z* = 4, and a half molecule is crystallographically independent (Figure 2a). A disorder model was applied to the peripheral benzene ring because a two-fold symmetry is imposed on the molecular axis. The O1-N1 bond length is 1.2824 (9) Å, being consistent with those of typical nitroxide groups [47,48,49]. The torsion angle around O1-N1-C2-C1 is 19.36(10)°, which is relatively small and guarantees the π conjugation throughout the *m*-phenylene-bridged bisnitroxide moiety. The biphenyl skeleton is largely twisted by the dihedral angle of 79.37(6)°, defined with the torsion C3-C4-C9-C10. This finding implies that the peripheral substituted benzene ring stabilizes the biradical by steric effects from the isopropyl groups (kinetic stabilization) rather than spin-delocalization effects due to the π-conjugation on the biphenyl core (thermodynamic stabilization). This notion is rationalized with practically no spin density found in the outer benzene ring, as indicated from the DFT calculation (see below).

The magnetic susceptibility measurement revealed an almost plateau profile in the *χ*_m_*T* vs. *T* plot (Figure 2b). A Curie–Weiss analysis, according to the equation *χ*_m_ = *C*/(*T* − *θ*), gave *C* = 1.03 cm^3^ K mol^−1^ and *θ* = −1.9 K from the data in the entire temperature range. The former indicates that each **iPr_3_BPBN** molecule behaved as a triplet molecule. The *M-H* curve (the inset) indicates that the saturation magnetization approaches 2 *μ*_B_, being consistent with the *S* =1 species at 1.8 K. A relatively slow saturation is ascribable to intermolecular antiferromagnetic coupling, as indicated with *θ* = −1.9 K.

The almost plateau profile in ca. 100–300 K of the *χ*_m_*T* vs. *T* plot suggests that the singlet-triplet energy gap is larger than the thermal energy of room temperature. The ground high-spin (*S* = 1) state is consistent with the spin-polarization mechanism [19,20,21,22,23,24]. Furthermore, the notably wide singlet-triplet gap is demonstrated, and this finding is accepted among related compounds such as **BPBN** [48] and many other derivatives [32,33,34,48,49,50,51]. The negative *θ* value is attributed to intermolecular antiferromagnetic coupling. The shortest intermolecular interatomic distances with respect to the N-O groups are found to be 4.5550 (11) Å for O•••N^†^, 4.7022(11) Å for N•••N^†^, and 4.7619 (11) Å for O••• O^†^ (the symmetry code of ^†^ is 1 − *x*, 2 − *y*, 1 − *z*).

### 3.2. Synthesis and Crystal Structure of ***1***

**Figure 3:**
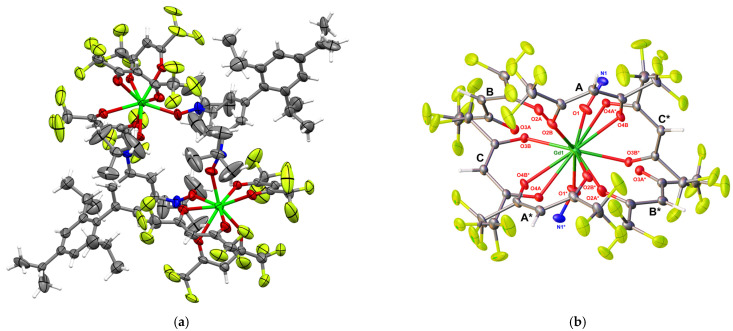
(**a**) X-ray crystal structure of **1** with thermal ellipsoids at the 50% probability level for non-hydrogen atoms. Solvent molecules are omitted. A half of disordered positions is shown. (**b**) An enlarged view of the Gd coordination environment to clarify disordered positions. Selective atomic numbering is also shown. The symmetry relation with (3/4 − *y*, 3/4 − *x*, 3/4 − *z*) is denoted with *. Color code: C, grey; N, blue; O, red; H, white; F, yellow; Gd, green.

According to the usual method known for lanthanide-nitroxide coordination compounds [13,14,15,16], combining **iPr_3_BPBN** and [Gd(hfac)_3_(H_2_O)_2_] with a molar ratio 1/1 in a dichloromethane-heptane mixed solvent afforded the polycrystalline product of [{Gd(hfac)_3_(μ-**iPr_3_BPBN**)}_2_] (**1**; Scheme 1). The composition was confirmed to be **1**•(CH_2_Cl_2_)_2_ by means of spectroscopic and X-ray diffraction analyses. The solvent molecules easily escape, giving rise to relatively large *R* factors. The formula was characterized as **1** by means of elemental analysis after desolvation. The molecular structure before desolvation is shown in Figure 3a. The space group is tetragonal *I*4_1_/*acd*, and a quarter molecule corresponds to an asymmetric unit. The Gd^3+^ ion is eight-coordinate. Two paramagnetic ligands doubly bridge two Gd^3+^ ions to form a 16-membered macrocycle.

A disorder was found as shown in Figure 3b. Two crystallograhically independent units are superposed here, and the six diketonate oxygen atoms must be assigned to three hfac groups. Accordingly, two sets from rings A, C, and B* and rings B, A*, and C* are given just the half occupancy each. Relatively long Gd-O distance was found for Gd1-O3A (2.657 (18) Å; Table S1, Supporting Information). It seems to be acceptable since there have been reports on the Gd-O bonds vary from 2.2 to 2.8 Å [52], and the steric congestion around Gd1 in **1** is responsible for the long distance.

The N-O bond length is 1.296 (10) Å, being typical of nitroxide complexes [13,14,16,47]. The coordination structure is best described as an approximate square antiprism from the SHAPE analysis [53], giving the CShM value of 1.431 to the ideal SAPR-8 for both disorder positions. The O1(rad)-Gd1 distance is 2.344 (8) Å and the O1(rad)-Gd1-O1(rad)^#^ angle is 84.0(4)°, where the symmetry operation code for ^#^ is (−*y* + 3/4, −*x* + 3/4, −*z* + 3/4). The coplanar nature across the *m*-phenylene and nitroxide groups is indicated with a relatively small torsion angle (30.3(13)° for O1-N1-C1-C2). Quite similarly to the case of uncoordinated **iPr_3_BPBN** (Figure 1a), the effective π conjugation is preferable to the spin polarization. The biphenyl skeleton is perfectly twisted by the dihedral angle of 88.9(9)°, defined with the two benzene planes. The isopropyl groups at the 2′- and 6′-positions work as a bulky protective group against possible deterioration at the benzene ring carrying two nitroxide groups.

The angular torsion around Gd-O-N-C(sp^2^) in **1** is 102.3(12)°. This angle has been proposed to be a convenient metric to describe the in-plane/out-of-plane geometry for a Gd^3+^ ion with respect to the nitroxide π* nodal plane. There have been a number of reports on ferromagnetic 4f-2p exchange couplings so far, and Kanetomo and co-workers comprehensively summarized the magneto-structural relationship [13]. The critical angle, *φ*_C_, where the sign of the exchange couplings changes from negative to positive is 40(2)°. Comparably large torsion is characterized in **1**, belonging to the “ferromagnetic region”.

**Scheme 3:**
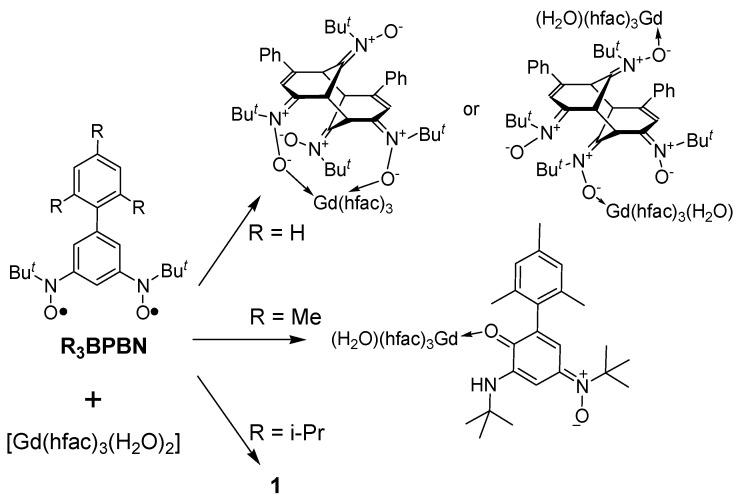
Reactions of BPBNs and [Gd(hfac)_3_(H_2_O)_2_], as a variation of substituents at the 2′,4′,6′-positions.

We should briefly summarize the protection of the radical group to explain the difference in reactivity. The mother **BPBN** underwent the [3+3] dimerization (Scheme 3, top) [54] and the trimethyl derivative (**Me_3_BPBN**) isomerized to a quinoneimine *N*-oxide (Scheme 3, middle) [34] in the presence of Gd^3+^. Namely, these ligands were transformed to diamagnetic ligands. Therefore, pursuing 2p-4f heterospin magnets involving triplet ligands requires biradicals more persistent against undesired dimerization and isomerization. The 2,4,6-triisopropylphenyl group in **iPr_3_BPBN** is supposed to play a role of steric protector against the spin-distributed *m*-phenylene group in a through-space manner. Eventually, we have found that **iPr_3_BPBN** is stable enough to survive during complexation reactions (Scheme 3, bottom).

**Scheme 4:**
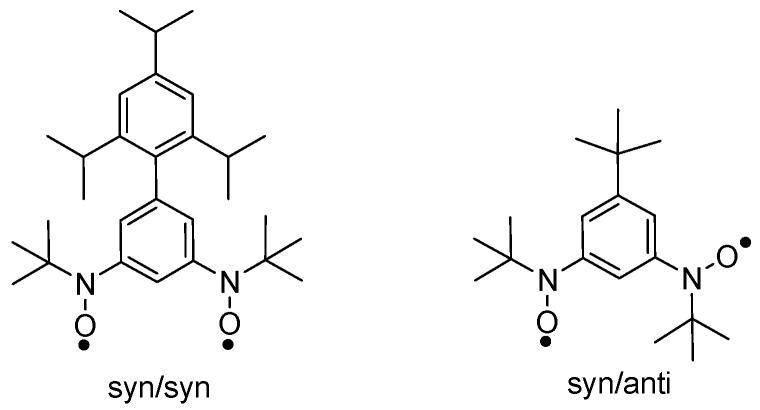
Syn/syn and syn/anti configurations of **iPr_3_BPBN** and **tBuPBN**, respectively.

Furthermore, we should also note the difference from the *tert*-butyl protecting group. Very recently, a linear chain system consisting of **tBuPBN** (Scheme 4) and Gd, Tb, Dy, Ho, or Er was reported [55]. Compared to that report, the present radical has a very bulky triisopropylphenyl group, so that the two nitroxide groups are directed in a syn/syn manner (Scheme 4, left) because of the steric congestion with bulky hfac groups located near the nitroxide oxygen atom. In the linear chain complex, the two nitroxide groups are configured in a syn/anti manner (Scheme 4, right). This picture provides us an idea on molecular and crystal design: the degree of bulkiness regulates a discrete macrocyclic molecule or an infinite one-dimensional polymer.

### 3.3. Magnetic Properties of ***1***

**Figure 4:**
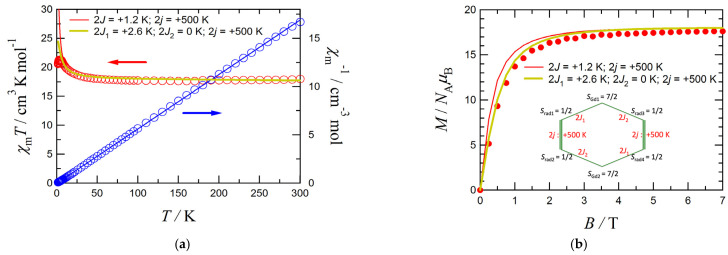
(**a**) Temperature dependence of *χ*_m_*T* and *χ*_m_^−1^ for **1** measured at 500 Oe. The Curie–Weiss best fit is drawn as a solid line on the *χ*_m_^−1^ data. Solid lines on the *χ*_m_*T* data stand for the simulation curves based on a model drawn in the inset of (**b**). The first model involves a condition *J* = *J*_1_ = *J*_2_. (**b**) *M-H* curve for **1** measured at 1.8 K. Solid lines stand for the simulation curves based on the same parameters as those of the solid lines in (**a**). The inset of (**b**) shows a schematic spin–spin coupling model.

The magnetic susceptibility was measured for **1**. As Figure 4a shows, the *χ*_m_*T* value increases on cooling from 300 K. A Curie-Weiss analysis gave *C* = 17.7 cm^3^ K mol^−1^ and *θ* = 0.66 K from the data above 6 K (a solid line on the *χ*_m_^−1^ data). The positive *θ* implies the presence of ferromagnetic interaction. Since the Gd ions and organic radicals can be treated as a spin-only species, the paramagnetic (high-temperature) limit of *C* would be 17.25 cm^3^ K mol^−1^ from two *S*_Gd_ = 7/2 and four *S*_rad_ = 1/2 with *g* = 2. The experimental value is larger than this paramagnetic limit. On the other hand, a calculation based on two *S*_Gd_ = 7/2 and two *S*_rad_ = 1 gives 17.75 cm^3^ K mol^−1^, which just coincides with the experimental value. The paramagnetic ligand contribution can be regarded as *S* = 1 in all of the temperature ranges. For *m*-phenylene bis(*tert*-butyl nitroxides), the exchange coupling constants larger than the thermal energy corresponding to room temperature can be found in the literature [32,33,48,49,50,51]. Therefore, the ferromagnetic interaction observed as an increase of *χ*_m_*T* can be assigned to 4f-2p exchange coupling through the direct Gd-nitroxide coordination bond. On further cooling from 6 to 1.8 K, the *χ*_m_*T* value seems to reach the limit. A saturation effect from the high-spin species may contribute as well to the *χ*_m_*T* drop.

The *M-H* curve at 1.8 K for **1** is shown in Figure 4b. The magnetization was almost saturated to be 17.6 *μ*_B_, which is very close to the theoretical ferromagnetic limit 18 *μ*_B_. However, the saturation seems to be somewhat slow, which is explained in terms of intermolecular antiferromagnetic interaction or when the ground state maximum spin multiplicity does not reach the highest (*S*_total_ = 9). Namely, a few possibilities are allowed to explain the magnetic properties. 

More quantitatively, a simulated *χ*_m_*T* vs. *T* curve was drawn on the MAGPACK program [56], using the spin Hamiltonian based on the molecular symmetry, $\hat{H}$ = −2*J*($\hat{S}$_Gd1_.$\hat{S}$_rad1_ + $\hat{S}$_Gd1_.$\hat{S}$_rad3_ + $\hat{S}$_Gd2_.$\hat{S}$_rad2_ + $\hat{S}$_Gd2_.$\hat{S}$_rad4_) − 2*j*($\hat{S}$_rad1_.$\hat{S}$_rad2_ + $\hat{S}$_rad3_.$\hat{S}$_rad4_). The simulation curve reproduced the experimental result except for the final *χ*_m_*T* plateau (Figure 4a, a solid line on the *χ*_m_*T* data). The adjustable parameter is unique with the intraligand interaction parameter 2*j* frozen to be +500 K (given from the DFT calculation, see below), and the 4f-2p exchange coupling parameter 2*J* was estimated to be +1.2 K. When 2*j* was variable in 300−1000 K, the simulation curves were confirmed to be hardly changed (Figure S4, Supporting Information), justifying that the calculated value was tentatively applied to the simulation. Furthermore, an approximate analysis seems to be possible, where the biradical is treated as a *S* = 1 species (Figure S5, Supporting Information). Though this treatment gave reasonable results, a two-centered biradical model seems to be more reliable than a one-centered model, because the consistency resides between the ESR results showing the spin-spin separation of *r* = 5.3 Å and the DFT results showing each spin mostly localized at each N-O group (see below).

Another possibility of the magnetic analysis requires a breakdown of the symmetry of the molecule on cooling. As mentioned in the Experimental section, the structure can also be solved in a space group *Ibca*, leading to *J*_1_ ≠ *J*_2_. A suitable spin Hamiltonian is assumed as $\hat{H}$ = −2*J*_1_($\hat{S}$_Gd1_.$\hat{S}$_rad1_ + $\hat{S}$_Gd2_.$\hat{S}$_rad2_) − 2*J*_2_($\hat{S}$_Gd1_.$\hat{S}$_rad3_ + $\hat{S}$_Gd2_.$\hat{S}$_rad4_) −2 *j*($\hat{S}$_rad1_.$\hat{S}$_rad2_ + $\hat{S}$_rad3_.$\hat{S}$_rad4_). It is difficult to simultaneously and precisely determine *J*_1_ and *J*_2_ owing to overparameterization. In this case, the final *χ*_m_*T* plateau can be ascribed to negligible *J*_2_; namely, the magnetic properties are described as practically two-molar *S* = 9/2 species. The theoretical *χ*_m_*T* maximum is 24.8 cm^3^ K mol^−1^. The simulation curve satisfactorily reproduced the experimental result except for the final *χ*_m_*T* plateau (Figure 4a, a solid line on the *χ*_m_*T* data). The 4f-2p exchange coupling parameter 2*J*_1_ was estimated to be +2.6 K. The latter possibility seems to be more likely, from viewing a better reproducibility in both *χ*_m_*T*-*T* and *M-H* behavior.

Quite similar macrocyclic motifs using 3d-2p spins, [{M(hfac)_2_(μ-**tBuPBN**)}_2_] (M = Mn [57,58], Co [59], Ni [60]; **tBuPBN** = 5-*tert*-butyl-1,3-phenylene bis(*tert*-butyl nitroxide), are known, and they showed all antiferromagnetic 3d-2p couplings, in sharp contrast to the 4f-2p case. The inverse trend can be understood by noting the following difference. This out-of-plane arrangement brings about SOMO(Mn, Co, or Ni 3d)–SOMO(O 2p) overlap for the former, whereas LUMO(Gd 5d)–SOMO(O 2p) overlap for the latter, where SOMO and LUMO stand for a singly-occupied molecular orbital and the lowest unoccupied (vacant) molecular orbital, respectively. The CT model, already mentioned in the Introduction section, completely holds for these systems upon the structures determined. It is also noteworthy that this concept has a much relation with that of the organic ferromagnet, β-NPNN (*p*-nitrophenyl nitroxyl nitroxide), proposed by Awaga et al. [61]. Andruh, Kahn, and co-workers have explained the ferromagnetic Gd^3+^-Cu^2+^ coupling similarly using a LUMO(Gd 5d)–SOMO(Cu 3d) overlap [62]. 

### 3.4. DFT Calculation on the Gd^3+^-**iPr_3_BPNB** System

**Figure 5:**
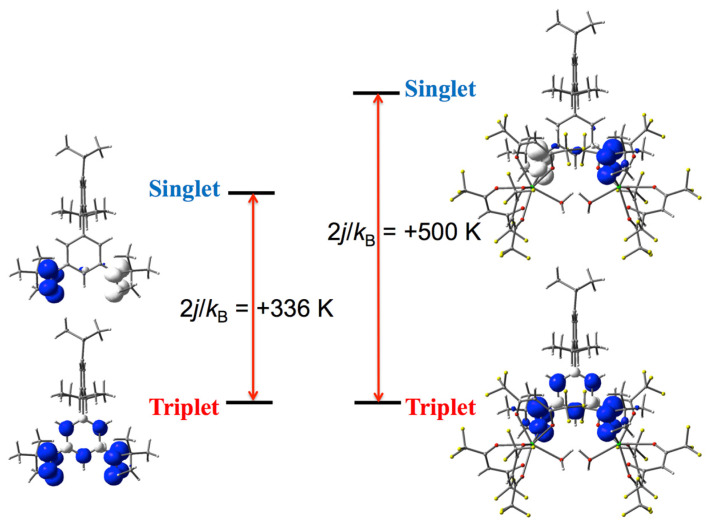
The relative energy levels of the triplet and singlet states for the uncoordinated ligand **iPr_3_BPBN** (left) and a model complex [{Y(hfac)_3_(H_2_O)}_2_(μ-**iPr_3_BPBN**)] (right). DFT calculation was performed on the geometries from the crystallography. Spin density surfaces are drawn at the 0.004 e^−^ Å^−3^ level with dark and light lobes for the positive and negative spin densities, respectively. For the atomic color code, see Figure 3. **iPr_3_BPBN**: The self-consistent field (SCF) energies are −1391.20031758 au with <*S*^2^> = 2.0002 and −1391.19929024 au with <*S*^2^> = 0.0708 for the triplet and singlet states, respectively. [{Y(hfac)_3_(H_2_O)}_2_(μ-**iPr_3_BPBN**)]: The SCF energies are -7265.92989889 au with <*S*^2^> = 2.0003 and −7265.92837108 au with <*S*^2^> = 0.0727 for the triplet and singlet states, respectively. For details, see Figures S6 and S7, Tables S2 and S3, Supporting Information.

The DFT calculation clarifies the ground multiplicity of the ligand when incorporated in complex **1**. Namely, the atomic coordinates of **iPr_3_BPBN** only were extracted from the experimental data of **1**•(CH_2_Cl_2_)_2_. The self-consistent field energies were calculated on the unrestricted B3LYP/6-311+G(d) level. As Figure 5 (left) shows, the ground state was triplet. The singlet-triplet gap +336 K is larger than the thermal energy of room temperature, being compatible with the experimental result. 

Interestingly, the presence of a trivalent metal ion enhances the intramolecular ferromagnetic exchange coupling, which seems to be a desirable factor to develop room-temperature triplet molecules. The experimentally determined coordinates were applied, except for the following: the paramagnetic Gd^3+^ ions were masked with diamagnetic Y^3+^ ions and the outer nitroxide oxygen site was replaced with a diamagnetic water molecule. The calculation on a model biradical complex [{Y(hfac)_3_(H_2_O)}_2_(μ-**iPr_3_BPBN**)] gave an improved singlet-triplet gap of +500 K (Figure 5, right), because a polarized canonical structure (>N^+•^–O^−^) contributes more than a neutral one (>N–O^•^) in the presence of cationic species near the oxygen atoms. This explanation is supported with the calculated spin densities at the nitrogen atoms; on average, 0.409 and 0.519 in **iPr_3_BPBN** and [{Y(hfac)_3_(H_2_O)}_2_(μ-**iPr_3_BPBN**)], respectively. 

**Figure 6:**
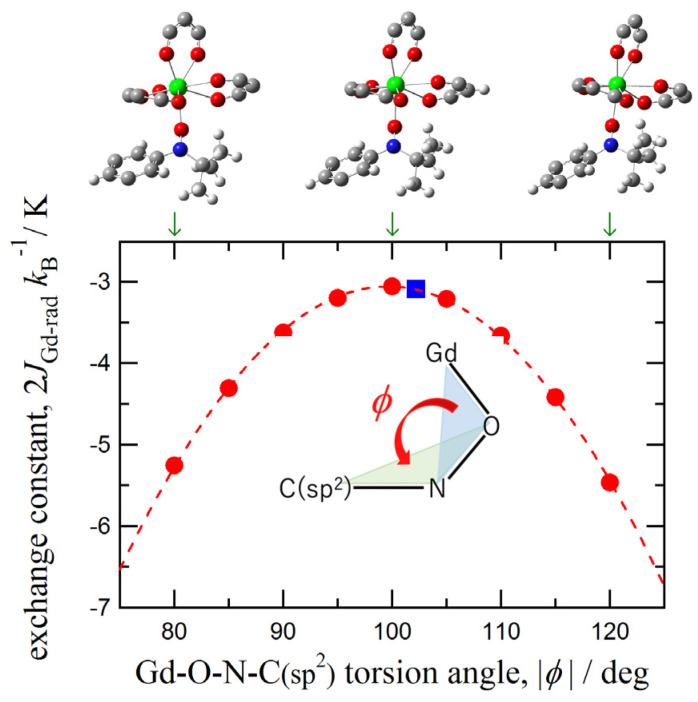
Computational magneto-structure relation by varying *φ* in **2**. Three model structures are drawn, where selected atoms are shown for clarity. For atomic color code, see Figure 5. A square symbol stands for the experimental structure. A broken line is drawn for a guide to the eye. Inset shows a schematic drawing of the definition of *φ*.

The validation of the DFT approach in lanthanide systems seems to be currently in progress, and Chibotaru noted in 2015 that in the case of Gd^3+^, “DFT methods may work” [63]. We attempted to estimate the exchange coupling constant *J*_Gd-rad_ in a two-centered virtual molecule, [Gd(hfac)_3_(H_2_O)(**iPr_3_BPBNH**)] (**2**), where the outer nitroxide oxygen site on the Gd^3+^ ion was replaced with a water molecule and the uncoordinated nitroxide group was masked with a diamagnetic hydroxylamine group (namely, **iPr_3_BPBNH**). The exchange coupling parameter was 2*J*_Gd-rad_/*k*_B_ = −3.09 K for **2** (Figures S8 and S9 and Table S4, Supporting Information). It is not astonishing to see a discrepancy compared to the experimental value on **1**, possibly because of oversimplification of a model including the masking technique. Nevertheless, we have found a qualitative trend in a simulation work for *J* as a function of *φ* in the virtual molecule (**2**). Model structures with 80° ≤ *φ* ≤ 120° were employed to avoid unrealistic interatomic proximity. As Figure 6 demonstrates, a twisted structure around *φ* = 100° maximizes *J*. The present calculation is quite reasonable, assuming that the ferromagnetic contribution is remarkable under strong twisting, because the resulting exchange interaction is expressed by the sum of the ferro- and antiferromagnetic terms (namely, *J* = *J*_F_ + *J*_AF_) [64]. This finding agrees with the proposed magneto-structural relation described in the Introduction section. 

## 4. Conclusions

A new ground triplet biradical, **iPr_3_BPBN**, was prepared, and its Gd^3+^ complex (**1**) has been characterized as a 2×2 macrocyclic compound. The molecular design works well; namely, (1) the ligand bridges metal ions, (2) the ligand possesses 2p-2p ferromagnetic exchange coupling, and (3) the 4f-2p ferromagnetic exchange coupling can be operational. Furthermore, (4) the highly reactive nature of the *S* = 1 biradical is suppressed by the introduction of bulky triisopropylphenyl. Bulky substituents will tune the dimensionality of the product and the sign and magnitude of 4f-2p exchange coupling. Compounds **iPr_3_BPBN** and **tBuPBN** are clarified to play a role of a potential triplet bridging ligand. Further explorations toward one-, two-, and three-dimensional architectures seem to be a promising strategy for the development of magnet-based materials.

## Figures and Tables

**Table 1:** Selected crystallographic parameters on **iPr_3_BPBN** and **1**•(CH_2_Cl_2_)_2_.

Compound	iPr_3_BPBN	1•(CH_2_Cl_2_)_2_
Formula	C_29_H_44_N_2_O_2_	C_45_H_49_Cl_2_F_18_GdN_2_O_8_
Crystal system	monoclinic	tetragonal
Space group	*C*2/*c*	*I*4_1_/*acd*
*a*/Å	10.6857 (5)	31.4028 (11)
*b*/Å	18.0214 (8)	31.4028 (11)
*c*/Å	13.8637 (6)	22.417 (3)
*β*/˚	96.604 (4)	90
*V*/Å^3^	2652.0 (2)	22106 (3)
*Z*	4	16
*d*_calcd_/g·cm^−3^	1.134	1.582
*μ* (MoKα)/mm^−1^	0.070	1.406
*R*_int_	0.0254	0.0450
*R*(*F*) ^a^, *R*_w_(*F*^2^) ^b^	0.0397, 0.1113	0.1287, 0.2991
GOF parameter	1.049	1.130
Δ*ρ*_max_, Δ*ρ*_min_/e Å^−3^	0.437, −0.233	1.876, −2.512
*T*/K	94 (2)	94 (1)
CCDC Reference	2192099	2077255

^a^*R* = Σ[|*F*_o_| − |*F*_c_|]/Σ|*F*_o_|; ^b^ *wR* = [Σ*w*(*F*_o_^2^ − *F*_c_^2^)/Σ*wF*_o_^4^]^1/2^.

## Data Availability

Not applicable.

## Supplementary Materials

The following is available online at: https://www.mdpi.com/article/10.3390/molecules27154930/s1. Selected spectroscopic data, simulation on *χ*_m_*T-T* and *M-H* for **1**, and details of the DFT calculation.
