# Supplementary material for: Ferromagnetic 2p-2p and 4f-2p Couplings in a Macrocycle from Two Biradicals and Two Gadolinium(III) Ions
Source: Molecules. 2022 Aug 2;27(15):4930. doi: 10.3390/molecules27154930 (PMC9370185; doi:10.3390/molecules27154930)
Supplement: Supplementary file 1 [file molecules-27-04930-s001.zip › Molecules-1826322-supplementary.pdf]

Supporting Information for

Ferromagnetic 2p-2p and 4f-2p Couplings in a Macrocycle from Two Biradicals and Two Gadolinium(III) Ions

Saki Ito, Toru Yoshitake, and Takayuki Ishida*

Department of Engineering Science, The University of Electro-Communications, Chofu, Tokyo 182-8585, Japan

**e-mail address: takayuki.ishida@uec.ac.jp*

Contents Page

Figure S1. ^{1}H NMR spectrum of the precursory bishydroxylamine p. S2

Figure S2. ^{13}C NMR spectrum of the precursory bishydroxylamine p. S3

Figure S3. FT-IR spectra of **iPr$_3$BPBN** and [{Gd(hfac)$_3$(μ-**iPr$_3$BPBN**)}$_2$] p. S4

Table S1. Selected geometric data of **1**•$(CH_2Cl_2)_2$. p. S5

Figures S4 and **S5**.

Simulation on $\chi_m T$-T and M-H for [{Gd(hfac)$_3$(μ-**iPr$_3$BPBN**)}$_2$] p. S6

Figure S6 and **Table S2**.

DFT calculation results on **iPr$_3$BPBN** pp. S7-8

Figure S7 and **Table S3.**

DFT calculation results on [{Y(hfac)$_3$(H_2O)}$_2$(μ-**iPr$_3$BPBN**)] pp. S9-12

Figure S8 and **Table S4.**

DFT calculation results on [Gd(hfac)$_3$(H_2O)(**iPr$_3$BPBNH**)] (**2**) pp. S13-15

Figure S9. Calculated spin densities of the nonet and septet states of **2.** p. S16

References p. S17

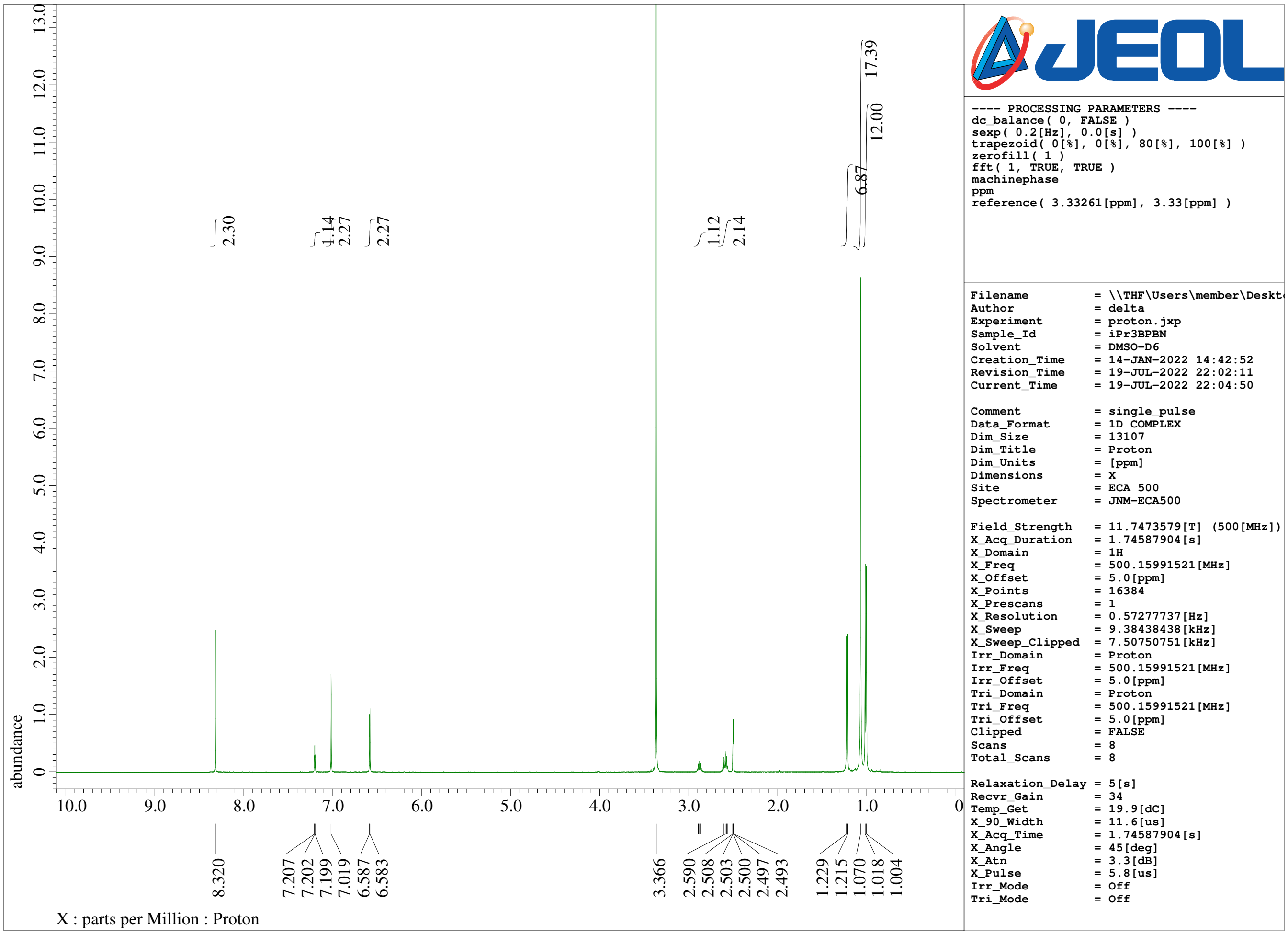

Figure S1. ^{1}H NMR spectrum of the bishydroxylamine as a precursor of **iPr$_3$BPBN** (2',4',6'-triisopropylbiphenyl-3,5-diyl bis(*t*-butyl nitroxide), measured in dimethyl sulfoxide-d_6 at room temperature. Chemical shifts are shown with respect to the residual solvent peak at 2.50 ppm.

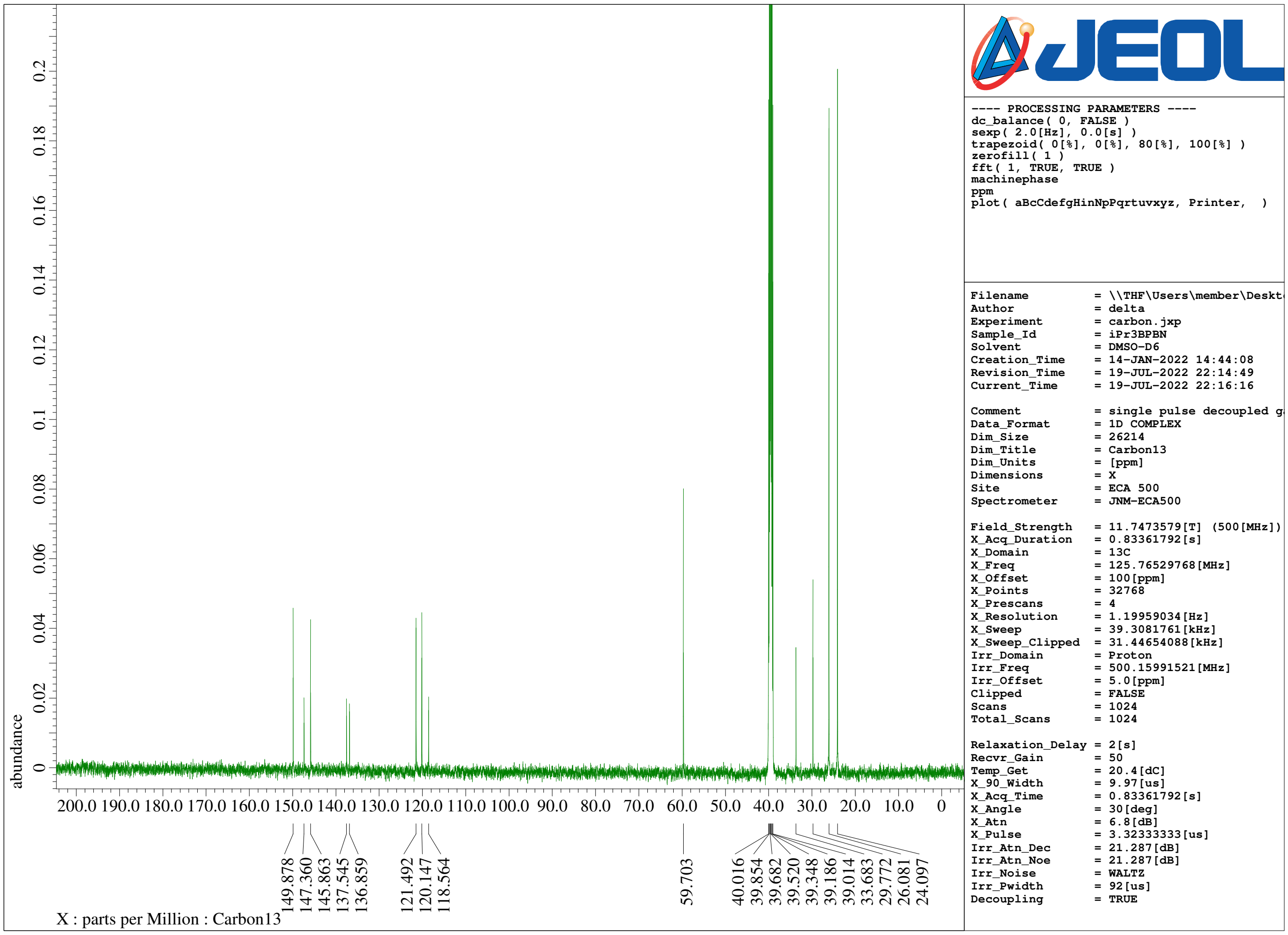

Figure S2. ^{13}C NMR spectrum of the bishydroxylamine as a precursor of **iPr$_3$BPBN**, measured in dimethyl sulfoxide-d_6 at room temperature. Chemical shifts are shown with respect to the solvent methyl signal at 39.52 ppm.

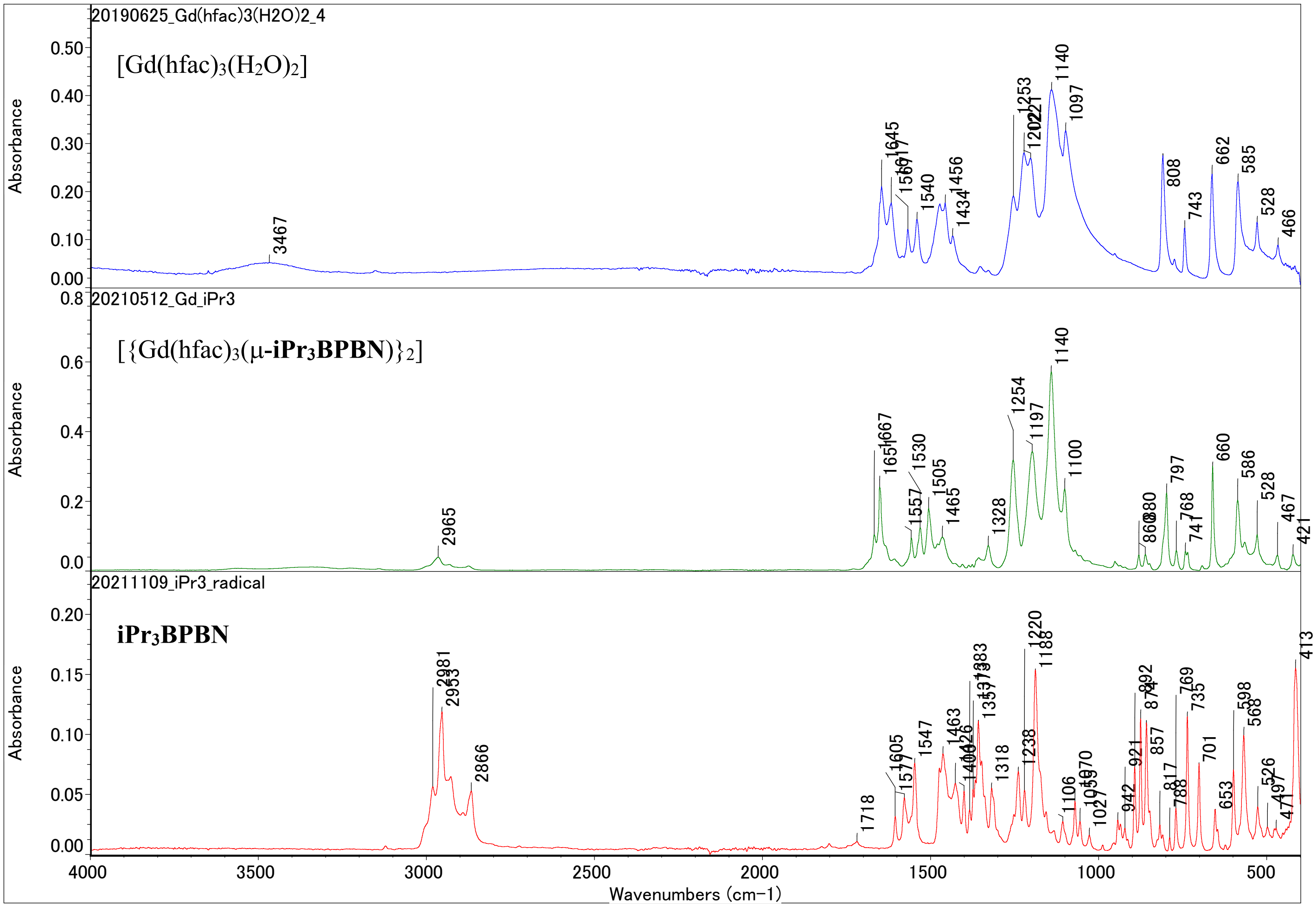

Figure S3. FT-IR spectra of neat samples for (top) [Gd(hfac)$_3$(H$_2$O)$_2$], (middle) [{Gd(hfac)$_3$(μ-**iPr$_3$BPBN**)}$_2$] (**1**), and (bottom) **iPr$_3$BPBN**.

Table S1. Selected geometrical data of **1**•$(CH_2Cl_2)_2$.[a)]

Compound	**1**•$(CH_2Cl_2)_2$
distance *d* / Å	
Gd1-O1	2.344(8)
Gd1-O1*	2.344(8)
Gd1-O2A	2.541(19)
Gd1-O2A*	2.541(19)
Gd1-O3A	2.657(18)
Gd1-O3A*	2.657(18)
Gd1-O3B	2.141(15)
Gd1-O3B*	2.141(15)
Gd1-O4A	2.371(17)
Gd1-O4A*	2.371(17)
Gd1-O4B	2.425(16)
Gd1-O4B*	2.425(16)
Gd1-O2B	2.166(16)
Gd1-O2B*	2.166(16)
O1-N1	1.296(10)
N1-C1	1.406(11)
N1-C5	1.501(16)

Compound	**1**•$(CH_2Cl_2)_2$
angle θ / °	
O1*-Gd1-O1	84.0(4)
O1*-Gd1-O2A	145.6(5)
O1*-Gd1-O2A*	96.0(6)
O1-Gd1-O2A*	145.6(5)
O1-Gd1-O2A	96.0(6)
O1-Gd1-O3A	70.3(5)
O1*-Gd1-O3A*	70.3(5)
O1-Gd1-O3A*	83.0(4)
O1*-Gd1-O3A	83.0(4)
O1-Gd1-O4A*	72.5(5)
O1*-Gd1-O4A	72.5(5)
O1*-Gd1-O4A*	144.5(5)
O1-Gd1-O4A	144.5(5)
O1*-Gd1-O4B*	80.4(5)
O1-Gd1-O4B	80.4(5)
O1*-Gd1-O4B	133.9(5)
O1-Gd1-O4B*	133.9(5)
O3B-Gd1-O1*	81.1(5)
O3B*-Gd1-O1*	75.1(5)
O3B-Gd1-O1	75.1(5)
O3B*-Gd1-O1	81.1(5)
O3B-Gd1-O4A	75.2(6)
O3B*-Gd1-O4A*	75.2(6)
O2B*-Gd1-O1	153.1(5)
O2B-Gd1-O1	109.7(5)
O2B-Gd1-O1*	153.1(5)
O2B*-Gd1-O1*	109.7(5)
O2B-Gd1-O4B	72.4(6)
O2B*-Gd1-O4B*	72.4(6)
O1-Gd1	144.9(7)
N1-C1	115.3(8)
N1-C5	116.2(9)

a) The symmetry operation code of * is $3/4 - y$, $3/4 - x$, $3/4 - z$.

(a) (b)

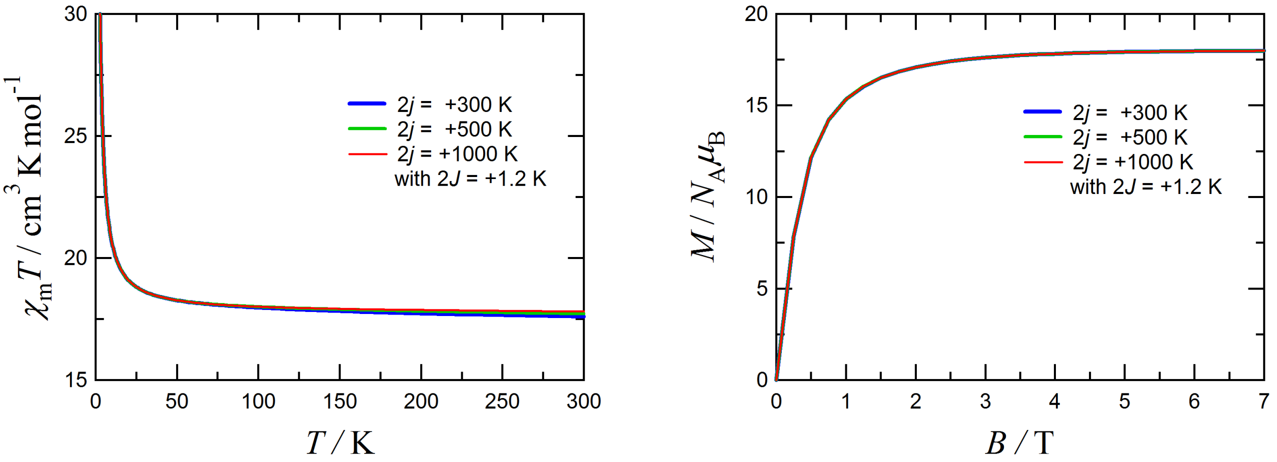

Figure S4. Simulated curves of the $\chi_{\mathrm{m}}T$ vs T (a) and M vs H (b) plots for [{Gd(hfac)$_3$(μ-**iPr$_3$BPBN**)}$_2$] (**1**). The adjustable parameter j is unique with the 4f-2p heterospin interaction parameter 2J frozen to be +1.2 K. The spin Hamiltonian utilized here was :

$$\hat{H} = -2J(\hat{S}_{\mathrm{Gd1}}\cdot\hat{S}_{\mathrm{rad1}} + \hat{S}_{\mathrm{Gd1}}\cdot\hat{S}_{\mathrm{rad3}} + \hat{S}_{\mathrm{Gd2}}\cdot\hat{S}_{\mathrm{rad2}} + \hat{S}_{\mathrm{Gd2}}\cdot\hat{S}_{\mathrm{rad4}}) - 2j(\hat{S}_{\mathrm{rad1}}\cdot\hat{S}_{\mathrm{rad2}} + \hat{S}_{\mathrm{rad3}}\cdot\hat{S}_{\mathrm{rad4}}).$$

(a) (b)

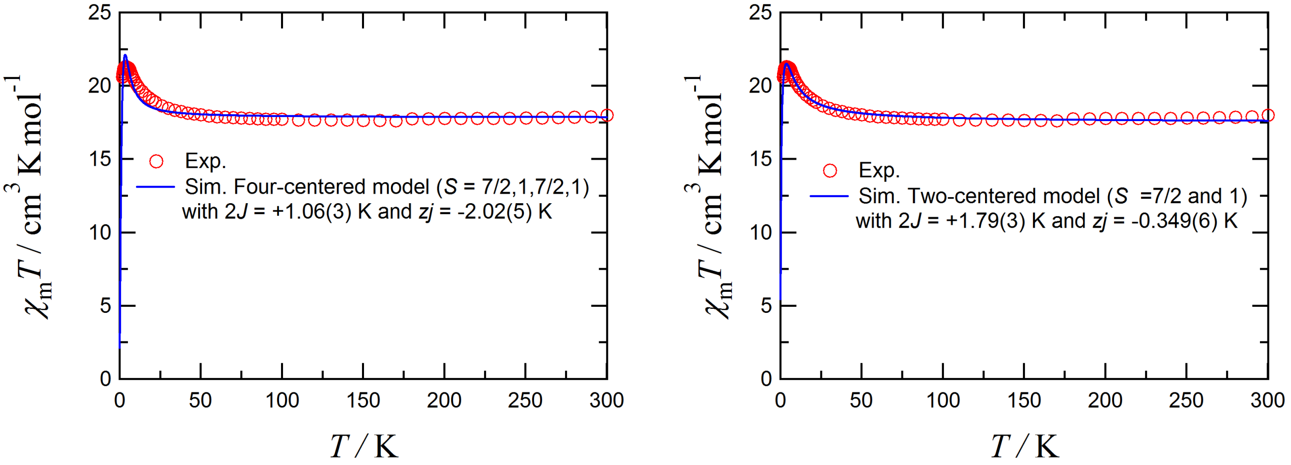

Figure S5. **iPr$_3$BPBN** is approximately treated as a S = 1 species, and a mean-field parameter (zj) was applied to the analysis. In (a), four spin centers (S = 7/2, 1, 7/2, and 1) are circularly arranged, and the nearest neighbors are coupled with a unique J. Intermolecular interactions correspond to zj. In (b), two spin centers (S = 7/2 and 1) are coupled with J. Intermolecular interactions as well as interaction between the counterparts are confined to zj.

Figure S6 and Table S2. Atomic numbering scheme and Mulliken atomic spin densities for the triplet and singlet states of **iPr$_3$BPBN**. DFT calculation was performed at the ub3lyp/6-311+g(d) level [S1].

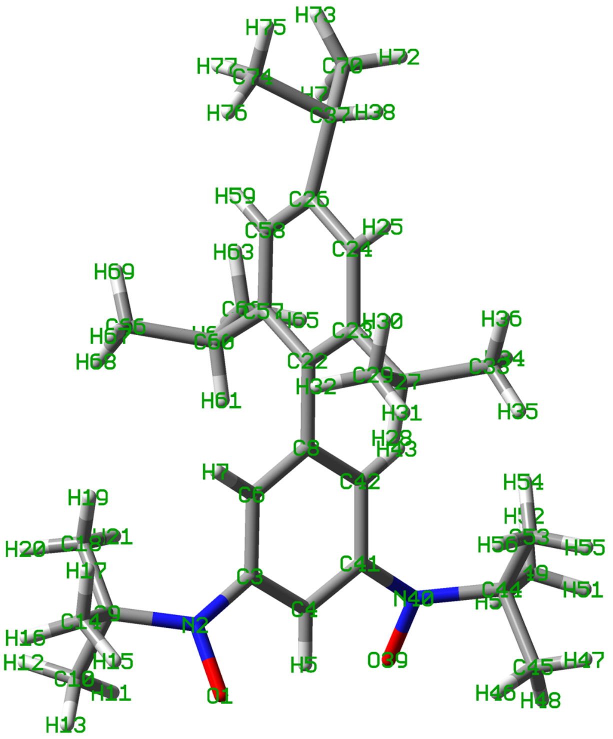

label	symbol	spin density	
		triplet	singlet
1	O	0.506882	0.514553
2	N	0.412135	0.405809
3	C	-0.142911	-0.045716
4	C	0.169098	0.000759
5	H	-0.007976	-0.000007
6	C	0.188855	0.011196
7	H	-0.011711	-0.000453
8	C	-0.025824	0.000372
9	C	-0.075718	-0.081737
10	C	0.025951	0.030074
11	H	-0.000433	-0.000387
12	H	-0.001249	-0.001139
13	H	0.000188	0.000167
14	C	0.009142	0.007639
15	H	-0.000230	-0.000300
16	H	0.000769	0.000712
17	H	-0.000823	-0.000891
18	C	0.022794	0.022207
19	H	-0.000003	-0.000411
20	H	-0.000113	-0.000540
21	H	-0.000619	-0.000475
22	C	-0.000462	0.000463
23	C	-0.007418	0.002688
24	C	0.000756	0.000462
25	H	-0.000131	-0.000001
26	C	-0.002226	-0.000223

27	C	0.009114	-0.001743
28	H	-0.000381	-0.000203
29	C	-0.000602	0.000066
30	H	-0.000010	-0.000005
31	H	-0.000051	-0.000006
32	H	-0.000114	-0.000111
33	C	-0.001037	0.000026
34	H	-0.000025	0.000028
35	H	-0.000029	0.000016
36	H	0.000018	0.000014
37	C	-0.000149	-0.000313
38	H	-0.000003	0.000000
39	O	0.506960	-0.514716
40	N	0.411874	-0.405344
41	C	-0.140753	0.042866
42	C	0.188129	-0.010322
43	H	-0.011726	0.000466
44	C	-0.076040	0.081988
45	C	0.026121	-0.030213
46	H	-0.000432	0.000389
47	H	-0.001236	0.001126
48	H	0.000191	-0.000170
49	C	0.008904	-0.007258
50	H	-0.000224	0.000293
51	H	0.000772	-0.000715
52	H	-0.000812	0.000881
53	C	0.022948	-0.022455
54	H	0.000005	0.000444
55	H	-0.000110	0.000540
56	H	-0.000598	0.000487
57	C	-0.007267	-0.002507
58	C	0.001371	-0.000636
59	H	-0.000131	0.000004
60	C	0.008718	0.001945
61	H	-0.000397	0.000211
62	C	-0.000450	-0.000023
63	H	-0.000009	0.000004
64	H	-0.000052	0.000009
65	H	-0.000131	0.000096
66	C	-0.001029	0.000135
67	H	-0.000039	-0.000054
68	H	-0.000028	-0.000022
69	H	0.000017	-0.000013
70	C	0.000016	-0.000034
71	H	-0.000003	-0.000004
72	H	-0.000003	0.000001
73	H	-0.000002	0.000000
74	C	-0.000005	0.000019
75	H	-0.000003	0.000000
76	H	0.000002	-0.000005
77	H	0.000003	-0.000003

Figure S7 and Table S3. Atomic numbering scheme and Mulliken atomic spin densities for the triplet and singlet states of [{Y(hfac)$_3$(H$_2$O)}$_2$(μ-**iPr$_3$BPBN**)]. DFT calculation was performed at the ub3lyp level with 6-311+g(d) for C, H, N, O, F and lanl2dz for Y [S1].

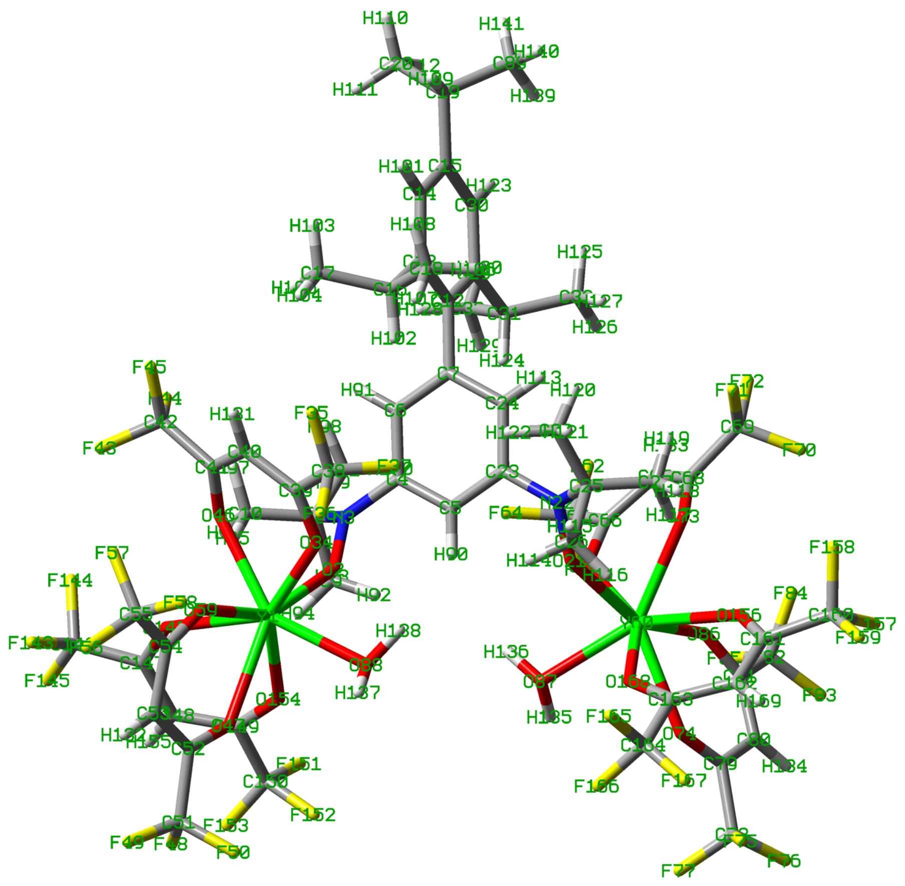

label	symbol	spin density	
		triplet	singlet
1	Y	0.030519	-0.028582
2	O	0.347883	-0.361528
3	N	0.518861	-0.518982
4	C	-0.105633	0.046268
5	C	0.154733	0.000289
6	C	0.193187	-0.002245
7	C	-0.078195	-0.000088
8	C	-0.036739	0.030891
9	C	0.031305	-0.031132
10	C	-0.026527	0.026164
11	C	0.022925	-0.017604
12	C	0.003557	0.000136
13	C	-0.002460	0.003318
14	C	0.001079	-0.000569
15	C	-0.002442	0.000307
16	C	0.005032	-0.002297
17	C	-0.000821	-0.000231
18	C	-0.000675	0.000724
19	C	-0.000449	0.000284

20	C	-0.000036	-0.000006
21	O	0.347979	0.361545
22	N	0.518241	0.518411
23	C	-0.104695	-0.046294
24	C	0.192858	0.002043
25	C	-0.037391	-0.031749
26	C	0.031489	0.031234
27	C	-0.025649	-0.025273
28	C	0.023090	0.017827
29	C	-0.002128	-0.003363
30	C	0.001752	0.000773
31	C	0.002018	0.001568
32	C	-0.000683	-0.000072
33	C	-0.000653	-0.000717
34	O	0.001100	-0.000747
35	F	0.000002	-0.000003
36	F	0.000023	-0.000018
37	F	-0.000016	-0.000039
38	C	-0.002108	0.002516
39	C	-0.004896	0.003290
40	C	0.006507	-0.005258
41	C	0.000049	0.002816
42	C	-0.000419	-0.002666
43	F	-0.000087	0.000068
44	F	0.000438	-0.000403
45	F	-0.000051	0.000038
46	O	0.002969	-0.004088
47	O	-0.000051	0.000079
48	F	0.000011	-0.000012
49	F	0.000034	-0.000035
50	F	-0.000004	0.000004
51	C	-0.000122	0.000114
52	C	-0.001105	0.001140
53	C	-0.000092	0.000048
54	C	0.001270	-0.001229
55	C	0.000570	-0.000521
56	F	0.000002	-0.000002
57	F	0.000012	-0.000014
58	F	-0.000003	0.000003
59	O	0.000434	-0.000437
60	Y	0.030961	0.028930
61	O	0.001312	0.000847
62	F	0.000004	0.000003
63	F	0.000016	0.000011
64	F	-0.000037	0.000041
65	C	-0.003758	-0.003768
66	C	-0.004692	-0.005731
67	C	0.005777	0.003776
68	C	0.001846	-0.001557
69	C	-0.000812	0.006587
70	F	-0.000082	-0.000062
71	F	0.000447	0.000422
72	F	-0.000054	-0.000037
73	O	0.002793	0.003750
74	O	-0.000033	-0.000060
75	F	0.000011	0.000012
76	F	0.000033	0.000034
77	F	-0.000003	-0.000004

78	C	-0.000082	-0.000083
79	C	-0.001234	-0.001261
80	C	0.000369	0.000389
81	C	0.000946	0.000987
82	C	0.000537	0.000464
83	F	0.000002	0.000002
84	F	0.000010	0.000009
85	F	-0.000005	-0.000005
86	O	0.000457	0.000449
87	O	0.003323	0.003700
88	O	0.003373	-0.003893
89	C	0.000050	0.000053
90	H	-0.012817	0.000058
91	H	-0.012876	-0.000210
92	H	-0.001416	0.001165
93	H	-0.001037	0.000960
94	H	-0.000459	0.000503
95	H	-0.001874	0.001927
96	H	0.001286	-0.001250
97	H	-0.000265	0.000267
98	H	0.000634	-0.000262
99	H	-0.000434	0.000861
100	H	-0.000796	0.001335
101	H	-0.000157	0.000017
102	H	0.000062	0.000431
103	H	-0.000032	0.000001
104	H	-0.000053	0.000053
105	H	-0.000012	0.000135
106	H	-0.000007	0.000019
107	H	-0.000038	0.000009
108	H	0.000022	-0.000013
109	H	-0.000002	0.000000
110	H	-0.000003	0.000002
111	H	0.000000	0.000014
112	H	0.000002	0.000003
113	H	-0.012878	0.000219
114	H	-0.001407	-0.001152
115	H	-0.001033	-0.000955
116	H	-0.000484	-0.000527
117	H	-0.001877	-0.001934
118	H	0.001316	0.001268
119	H	-0.000302	-0.000304
120	H	0.000647	0.000219
121	H	-0.000432	-0.000868
122	H	-0.000745	-0.001446
123	H	-0.000156	-0.000021
124	H	0.000101	-0.000370
125	H	-0.000030	0.000001
126	H	-0.000047	-0.000046
127	H	-0.000018	-0.000117
128	H	-0.000017	0.000014
129	H	-0.000036	-0.000015
130	H	0.000022	0.000012
131	H	-0.000091	0.000037
132	H	-0.000003	0.000003
133	H	-0.000090	-0.000040
134	H	-0.000001	-0.000001
135	H	0.000026	0.000014

136	H	0.000014	-0.000302
137	H	0.000034	-0.000025
138	H	0.000046	0.000327
139	H	0.000003	0.000008
140	H	-0.000005	-0.000003
141	H	-0.000001	0.000001
142	O	-0.000508	0.000429
143	F	0.000009	-0.000009
144	F	-0.000008	0.000004
145	F	-0.000003	0.000003
146	C	0.000418	-0.000315
147	C	0.001710	-0.001658
148	C	-0.000621	0.000737
149	C	-0.000430	0.000258
150	C	0.000190	-0.000351
151	F	0.000074	-0.000077
152	F	-0.000026	0.000025
153	F	0.000014	-0.000015
154	O	-0.000721	0.000523
155	H	0.000006	-0.000007
156	O	-0.000519	-0.000422
157	F	0.000008	0.000008
158	F	-0.000004	-0.000001
159	F	-0.000003	-0.000004
160	C	0.000259	0.000195
161	C	0.001705	0.001731
162	C	-0.000286	-0.000570
163	C	-0.000636	-0.000361
164	C	0.000585	0.000722
165	F	0.000059	0.000061
166	F	-0.000025	-0.000024
167	F	0.000013	0.000014
168	O	-0.000827	-0.000626
169	H	0.000006	0.000007

Figure S8 and Table S4. Atomic numbering scheme and Mulliken atomic spin densities for the nonet and septet states of [Gd(hfac)$_3$(H$_2$O)(**iPr$_3$BPBNH**)] (**2**). DFT calculation was performed at the ub3lyp level with 6-311+g(d) for C, H, N, O, F and sdd for Gd [S1].

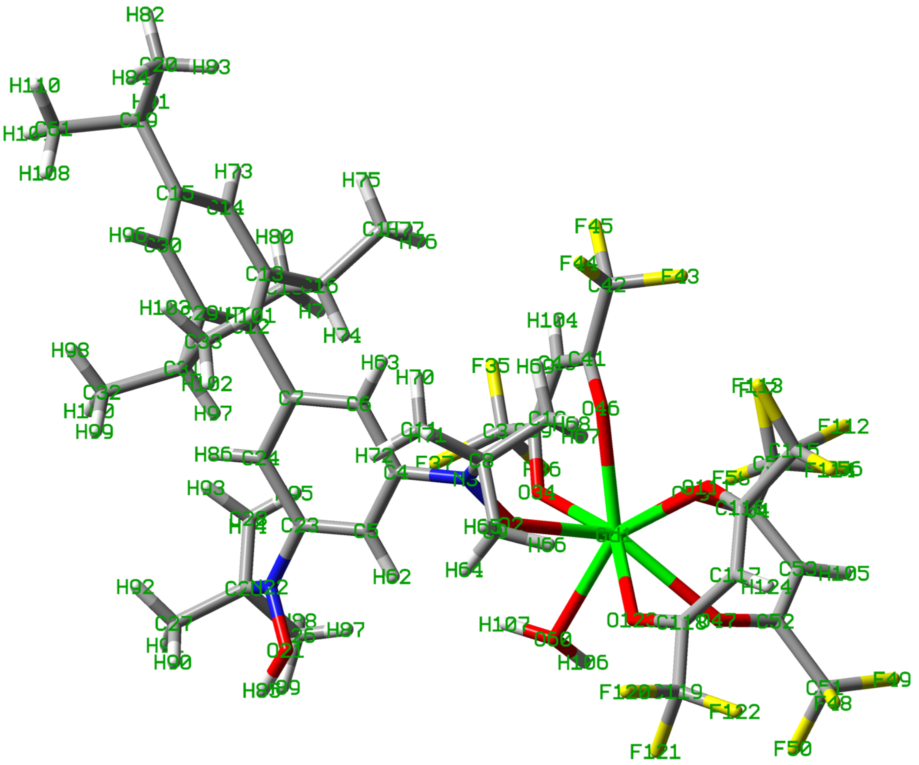

label	symbol	spin density	
		nonet	septet
1	Gd	7.059999	7.024140
2	O	0.325027	-0.330321
3	N	0.580836	-0.580015
4	C	-0.102973	0.102022
5	C	0.095142	-0.095270
6	C	0.107015	-0.106863
7	C	-0.044211	0.043922
8	C	-0.072596	0.073241
9	C	0.034530	-0.034476
10	C	0.019978	-0.018891
11	C	-0.000113	-0.000001
12	C	0.001689	-0.001715
13	C	-0.000743	0.000691
14	C	0.000953	-0.000927
15	C	-0.001450	0.001459
16	C	0.001738	-0.001778
17	C	0.000606	-0.000536
18	C	-0.000361	0.000344
19	C	-0.000306	0.000305
20	C	-0.000023	0.000025
21	O	-0.002458	0.002456
22	N	-0.006164	0.006056

23	C	-0.044762	0.044615
24	C	0.108710	-0.108479
25	C	0.000975	-0.001030
26	C	-0.001284	0.001283
27	C	-0.001009	0.001025
28	C	0.002505	-0.002465
29	C	-0.001245	0.001227
30	C	0.000796	-0.000778
31	C	0.003063	-0.003054
32	C	-0.000289	0.000277
33	C	-0.000410	0.000414
34	O	-0.005245	-0.005998
35	F	-0.000072	-0.000070
36	F	-0.000016	0.000033
37	F	-0.000057	0.000186
38	C	-0.004457	0.004034
39	C	0.003858	-0.010719
40	C	0.000805	0.001949
41	C	-0.012183	0.017577
42	C	0.003631	-0.004380
43	F	-0.000059	0.000072
44	F	0.000620	-0.000582
45	F	-0.000111	-0.000021
46	O	-0.011883	-0.002916
47	O	-0.004559	-0.004383
48	F	0.000035	0.000035
49	F	-0.000012	-0.000069
50	F	0.000038	0.000049
51	C	0.000253	-0.000319
52	C	0.002815	0.002606
53	C	-0.000793	-0.001520
54	C	-0.000540	-0.000370
55	C	-0.001217	-0.000776
56	F	-0.000090	-0.000098
57	F	0.000065	0.000065
58	F	0.000048	0.000039
59	O	-0.009398	-0.012714
60	O	-0.000747	-0.002668
61	C	0.000001	-0.000001
62	H	-0.004981	0.004945
63	H	-0.005842	0.005839
64	H	-0.000258	0.000276
65	H	0.000431	-0.000407
66	H	-0.000180	0.000224
67	H	0.001357	-0.001413
68	H	0.001142	-0.001148
69	H	-0.000388	0.000405
70	H	0.000444	-0.000446
71	H	-0.000467	0.000469
72	H	-0.000860	0.000857
73	H	-0.000093	0.000093
74	H	-0.000167	0.000183
75	H	-0.000006	0.000007
76	H	-0.000055	0.000053
77	H	-0.000095	0.000098
78	H	-0.000033	0.000033
79	H	-0.000018	0.000019
80	H	0.000014	-0.000014

81	H	0.000000	0.000000
82	H	-0.000002	0.000002
83	H	-0.000002	0.000002
84	H	0.000000	0.000000
85	H	-0.000280	0.000281
86	H	-0.007097	0.007086
87	H	-0.000100	0.000097
88	H	-0.000163	0.000164
89	H	0.000011	-0.000011
90	H	0.000021	-0.000021
91	H	0.000070	-0.000069
92	H	0.000032	-0.000032
93	H	0.000192	-0.000192
94	H	0.000299	-0.000299
95	H	0.000387	-0.000378
96	H	-0.000076	0.000075
97	H	0.000227	-0.000224
98	H	-0.000013	0.000013
99	H	-0.000018	0.000018
100	H	-0.000059	0.000058
101	H	0.000033	-0.000033
102	H	-0.000004	0.000004
103	H	0.000013	-0.000013
104	H	-0.000067	0.000007
105	H	0.000057	0.000072
106	H	-0.000445	-0.000445
107	H	0.000071	-0.000109
108	H	-0.000003	0.000003
109	H	-0.000001	0.000001
110	H	-0.000001	0.000001
111	O	-0.005086	-0.005644
112	F	0.000000	-0.000003
113	F	0.000070	-0.000018
114	F	-0.000067	-0.000063
115	C	-0.000369	-0.000416
116	C	0.003499	0.000905
117	C	0.000473	-0.000153
118	C	-0.003611	-0.001753
119	C	0.001810	-0.003023
120	F	0.000056	-0.000014
121	F	-0.000042	0.000037
122	F	-0.000056	-0.000053
123	O	-0.003612	-0.001875
124	H	0.000015	-0.000004

Figure S9. Calculated spin densities of the nonet and septet states of **2**.

Nonet

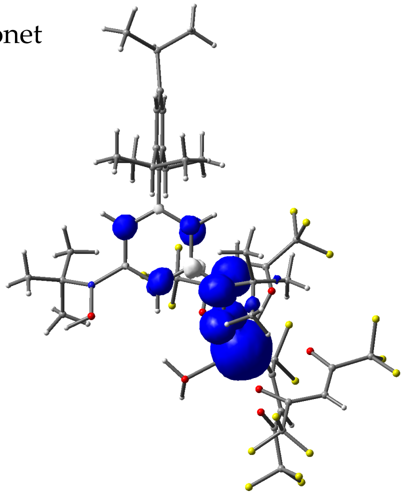

Septet

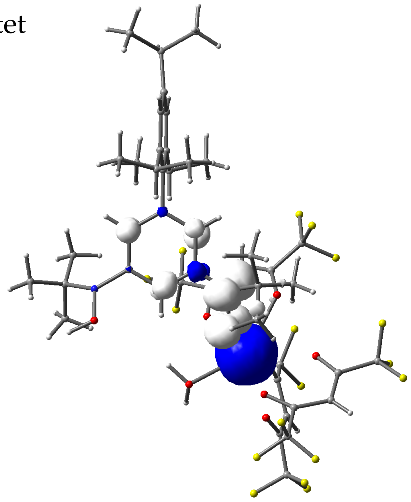

Note: The SCF energies were –5056.91058803 au with $\langle S^2 \rangle$ = 20.0001 and –5056.91062710 au with $\langle S^2 \rangle$ = 12.0125 for the nonet and septet states, respectively.

References

(S1) *Gaussian 16, Revision C.01*, Frisch, M. J.; Trucks, G. W.; Schlegel, H. B.; Scuseria, G. E.; Robb, M. A.; Cheeseman, J. R.; Scalmani, G.; Barone, V.; Petersson, G. A.; Nakatsuji, H. *et al.* Gaussian, Inc., Wallingford CT, 2016.
